# A novel loss-of-function mutation of *GATA3* (p.R299Q) in a Japanese family with Hypoparathyroidism, Deafness, and Renal Dysplasia (HDR) syndrome

**DOI:** 10.1186/s12902-015-0065-7

**Published:** 2015-10-30

**Authors:** Tetsuji Okawa, Masanori Yoshida, Takeshi Usui, Takahiro Kudou, Yasumasa Iwasaki, Kazuki Fukuoka, Norio Takahashi, Yuka Uehara, Yutaka Oiso

**Affiliations:** Department of Endocrinology and Diabetes, Nagoya Ekisaikai Hospital, 4-66 Shounen-cho, Nakagawawa-ku, Nagoya 454-8502 Japan; Clinical Research Institute, National Hospital Organization Kyoto Medical Center, Kyoto, 612-8555 Japan; Laboratory of Protein Informatics, Research Center for State-of-the-Art Functional Protein Analysis, Institute for Protein Research, Osaka University, Suita, 565-0871 Japan; Health Care Center, Kochi University, Kochi, 780-8520 Japan; Department of Endocrinology and Diabetes, Nagoya University Graduate School of Medicine, Nagoya, 466-8550 Japan

**Keywords:** Hypoparathyroidism, Deafness, and renal dysplasia (HDR) syndrome, Zinc finger, GATA3, Heterozygous missense mutation, Structure prediction model, Luciferase reporter assay

## Abstract

**Background:**

Hypoparathyroidism, deafness, and renal dysplasia (HDR) syndrome is a rare autosomal dominant disorder caused by mutations in the zinc finger transcription factor gene, *GATA3*. GATA3 has 2 zinc finger domains, which play an important role in the increase in target gene transcription activity.

**Case presentation:**

A 50-year-old woman and her 27-year-old daughter were followed up because of hypoparathyroidism. They had bilateral sensorineural deafness. Abdominal computed tomography scanning revealed renal dysplasia in the mother, but no renal anomaly in the daughter. Direct sequencing of *GATA3* gene revealed a novel heterozygous missense mutation at codon 299 (p.R299Q) in exon 4. This mutation is located at the junction between the 2 zinc fingers. The structure prediction showed that it caused a conformation change in this junction area, affecting the spatial position of the zinc fingers. Additionally, a more marked conformation change was observed in the N-terminal zinc finger region compared to that in the C-terminal region. Functional analysis of this mutant protein using an *in vitro* luciferase reporter assay system confirmed that the mutation abolished the enhancing effects of wild-type GATA3 on the promoter activity of the consensus GATA responsive element and that of human *PTH* gene.

**Conclusion:**

We identified a novel R299Q mutation in *GATA3* in a Japanese family with HDR syndrome. We confirmed that R299Q is a loss-of-function mutation, due to the extensive conformational change in the zinc fingers of GATA3.

## Background

Hypoparathyroidism, deafness, and renal dysplasia (HDR) syndrome is a rare hereditary autosomal dominant disorder [1]. Haploinsufficiency of *GATA3* gene, located on chromosome 10p15, is responsible for HDR syndrome [2, 3]. Human *GATA3* consists of 6 exons that span 20 kb of genomic DNA and encode 444 amino acids [4]. GATA3 is a dual zinc finger transcription factor that binds to the consensus motif (A/T)GATA(A/G) on the target gene promoter region [5–7]. This transcription factor plays a pivotal role in differentiation in the parathyroid gland, inner ear, kidney, thymus, and central nervous system [8, 9]. Patients with HDR syndrome mainly present with a triad of clinical symptoms, viz., primary hypoparathyroidism, sensorineural deafness, and renal dysplasia with variable presentation even within individuals with the same mutation [10, 11]. On the other hand, GATA3 also serves as a master regulator of the differentiation of T helper type 2 cells [5, 6], but no immune deficient symptom has been reported in patients with HDR syndrome.

Here, we report a novel mutation of *GATA3* in a Japanese family with HDR syndrome, presenting with different renal phenotypes. We analyzed the 3-dimensional structure and transcriptional function of this mutant GATA3.

## Case presentation

**Fig. 1:**
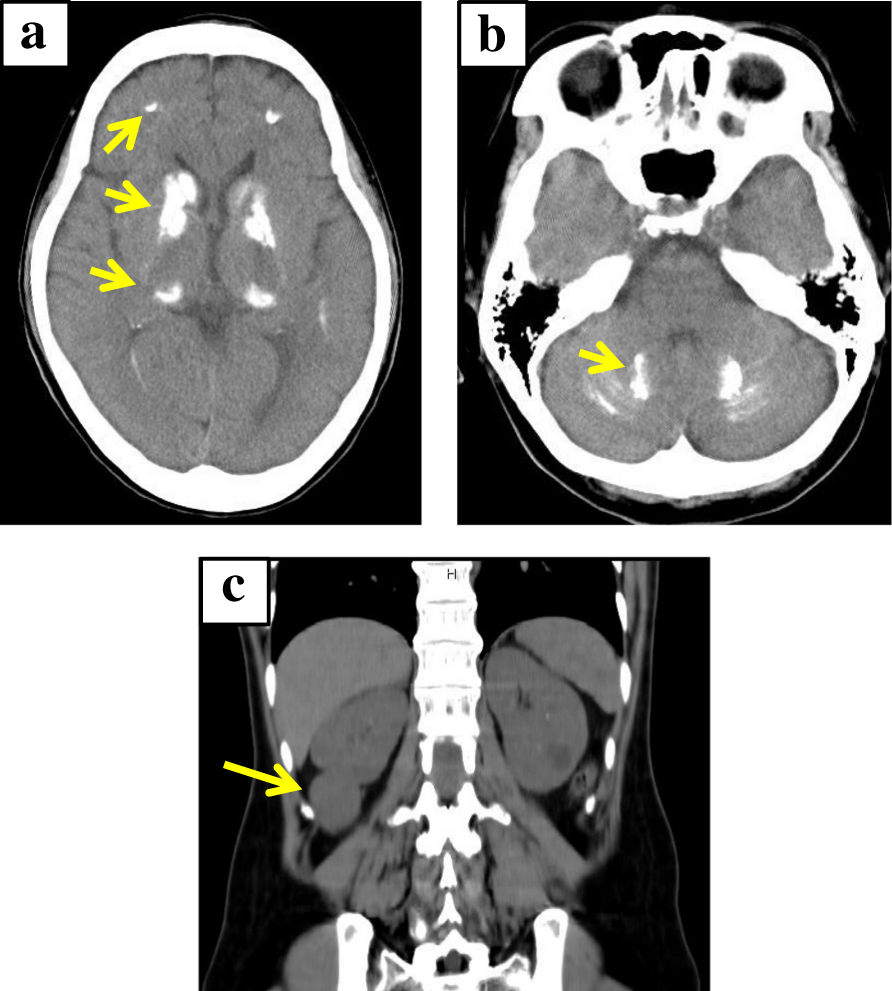
Plain head and abdominal computed tomography (CT) scans of the proband. **a**, **b**. Head CT. Severe calcification was observed in the basal ganglia, thalamus, deep white matter (**a**), and cerebellum (**b**) (arrow). **c**. Abdominal CT. Renal dysplasia can be seen on the right side (arrow)

A 50-year-old Japanese woman had been followed up at our hospital because of hypoparathyroidism. At the age of 33 years, the patient had suddenly experienced a general convulsion with loss of consciousness and was taken to the emergency room of our hospital. Upon arrival, she regained consciousness. She was positive for the Trousseau sign. Blood examination showed severe hypocalcemia (5.2 mg/dL) and hyperphosphatemia (4.7 mg/dL) with a relatively low level of intact PTH (7 pg/mL). Serum creatinine and blood urea nitrogen levels were normal (0.6 mg/dL and 9.1 mg/mL, respectively) with no other electrolyte disorder. Percentage of tubular reabsorption of phosphate was 98.4 % (reference range: 81–90 %). Anterior pituitary hormone levels and thyroid and adrenal functions were normal. She was diagnosed with idiopathic hypoparathyroidism, and calcium lactate and 1α-hydroxyvitamin D_3_ treatment were initiated. After calcium lactate was discontinued, administration of 2 μg of 1α-hydroxyvitamin D_3_ was sufficient to maintain her serum calcium level within the normal range. Head computed tomography (CT) scanning showed severe brain calcification. CT images at the age of 47 years are shown in Fig. 1a and b; calcification was observed in the bilateral basal ganglia, thalamus, cerebellum, and deep white matter. She had mild juvenile cataracts in both eyes. Fluorescence in situ hybridization did not indicate 22q11.2 syndrome, as no microdeletion was observed in the 22q11.2 region (data not shown). Hearing disturbance progressed gradually; an audiogram performed at the age of 41 years indicated bilateral sensorineural deafness (right: 47 dB, left: 55 dB). Abdominal CT revealed renal dysplasia on the right side (Fig. 1c). These clinical findings were consistent with HDR syndrome.

This patient’s 27-year-old daughter also had hypoparathyroidism, which was incidentally identified when she visited our hospital for a common cold at the age of 14 years; blood examination showed hypocalcemia (5.7 mg/dL) and hyperphosphatemia (6.8 mg/dL) with a low level of intact PTH (9 pg/mL). Thereafter, she had received 1 μg of 1α-hydroxyvitamin D_3_. She had a moderate hearing disturbance (right: 56 dB, left: 47 dB). Unlike her mother, faint calcifications in the basal ganglia and no renal abnormalities were observed.

**Fig. 2:**
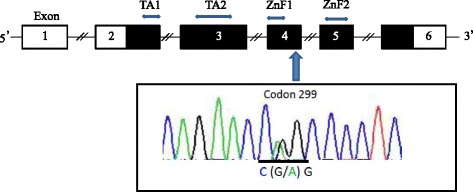
Direct sequencing of *GATA3*. Upper panel shows the genomic structure of *GATA3*, consisting of 6 exons (indicated by boxes). The black and white boxes denote the coding regions and the untranslated regions, respectively. *GATA3* encodes a protein with 2 transactivating domains (TA1 and TA2) and 2 zinc finger domains (N-terminal zinc finger [ZnF1] and C-terminal zinc finger [ZnF2]). ZnF1 is encoded by exon 4 and ZnF2 is encoded by exon 5. The arrow indicates the mutation site. The lower panel shows the nucleotide sequences around codon 299 in exon 4 of *GATA3*

Sequencing analysis was performed for all coding regions, including the adjacent intronic regions, of *GATA3* from the peripheral blood of the mother. A novel heterozygous p.R299Q (CGG > CAG) missense mutation was found in exon 4 (Fig. 2). This codon 299 amino acid of GATA3 was located in the liker region, consisting of 30 amino acids, between 2 consecutive zinc finger domains. These zinc fingers are important for the increase in the transcriptional activity of GATA3 target genes.

**Fig. 3:**
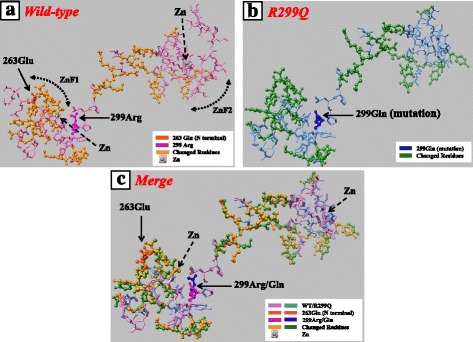
Three-dimensional structural modeling of wild-type and mutant GATA3 proteins. **a**, Wild-type GATA3 protein. **b**, GATA3 mutant protein (R299Q). **c**, Merge of (**a**) and (**b**). Residues 263–347 are shown. This region includes ZnF1 (residues 264–288) and ZnF2 (residues 318–342). The arrows indicate residues 263 (N-terminal) and 299. Residues with a significant conformational change by R299Q mutation are color-coded (bright yellow [wild-type] and green [mutant])

We analyzed the 3-dimensional structural models of wild-type (GATA3/WT) and mutant (GATA3/R299Q) GATA3 using the Sequence to Function Annotation Server (SFAS) provided by the Protein Data Bank Japan (PDBj: http://pdbj.org/) [12]. The structure of both proteins was compared and visualized by UCSF Chimera, developed by the Resource for Biocomputing, Visualization, and Informatics (RBVI) (http://www.cgl.ucsf.edu/chimera/) [13]. The protein conformation showing the 2 zinc fingers is presented in Fig. 3. This mutation altered the spatial position of the 2 zinc fingers through a conformation change in the linker between the zinc fingers. A more marked conformational change was observed in the position of the N-terminal zinc finger (ZnF1; residues 264–288) than in the C-terminal zinc finger (ZnF2; residues 318–342), probably because the Arg-299 residue is located near the zinc ion of ZnF1 (Fig. 3a). The conformational change also extended into a part of ZnF2. This extensive conformational change probably affects the biological function of GATA3.

**Fig. 4:**
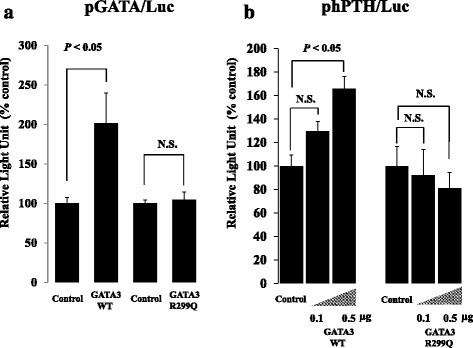
Functional analysis of wild-type and mutant GATA3 proteins using a　luciferase reporter assay system. **a**, Effects of the overexpression of wild-type and mutant GATA3 on the transcription activity of the heterologous promoter. Mutant GATA3 overexpression abolished the enhancing effects of wild-type GATA3 on the promoter containing the consensus GATA responsive element (pGATA/Luc). **b**, Effects of the overexpression of wild-type and mutant GATA3 on human *PTH* gene expression. Wild-type GATA3 overexpression significantly enhanced the transcription activity of human *PTH* (phPTH/Luc), whereas this stimulatory effect was not observed with mutant GATA3 overexpression. Data are expressed as folds increase over control group. *P* < 0.05 vs. corresponding control

The transcriptional function of GATA3/R299Q was evaluated using a luciferase reporter assay system. The transcription activity of pGATA/Luc with a consensus GATA responsive element significantly increased when the GATA3/WT expression vector was transfected, whereas this enhancing effect was completely lost in the GATA3/R299Q vector-introduced cells (Fig. 4a). This result indicated that GATA3/R299Q severely affected the transcription activity of various GATA3-regulated genes. We next examined the effects of GATA3/WT and GATA3/R299Q on the promoter activity of the *PTH* gene. GATA3/WT overexpression significantly enhanced the transcription activity of human *PTH* in a dose-dependent manner, whereas this stimulatory effect was not observed with GATA3/R299Q overexpression (Fig. 4b). These data suggested that the GATA3/R299Q protein failed to increase the *PTH* gene expression. The extensive conformational change of GATA3/R299Q most likely reduces the transcriptional activity of GATA3 target genes associated with fetal differentiation of the organs affected in HDR syndrome, such as the parathyroid gland, inner ear, and kidney.

## Method

### Preparation of DNA and sequence analysis

DNA was extracted from peripheral blood leukocytes using standard methods. All PCR-amplified exons and splice junctions of *GATA3* from the proband were directly sequenced [14], after permission was obtained from the patient and the ethical committees of Nagoya Ekisaikai Hospital and National Hospital Organization Kyoto Medical Center.

### Plasmid construction

Full-length human *GATA3* cDNA was kindly provided by RIKEN BioResource Center (Tsukuba, Japan) as a plasmid [15]. A GATA3 expression vector (GATA3/WT) was constructed by insertion of the human *GATA3* cDNA into a pRc/Rous sarcoma virus (RSV) vector (Invitrogen, San Diego, CA). Specific primers used for PCR were as follows: forward 5′- GAGGCCATGGAGGTGACGGC-3′, reverse 5′-TCTAACCCATGGCGGTGACC-3′; mutant construct (GATA3/R299Q) was made from GATA3/WT by a site-directed mutagenesis technique using the primers as follows: forward 5′-GGACAGAACCAGCCCCTCATTA-3′, reverse 5′-TAATGAGGGGCTGGTTCTGTCC-3′. For the luciferase assay, 2 luciferase reporter plasmids were used: pGATA/Luc, containing the consensus GATA binding element (LR-2103, Signosis, Santa Clara, CA), and phPTH/Luc containing the 5′-promoter region of human *PTH* (−2000/+54; +1 designates the transcription start site) [16].

### Cell culture and transfection

HepG2 cells were maintained in T_75_ culture flasks using DMEM culture medium supplemented with 10 % (v/v) fetal bovine serum, penicillin (100 U/ml), and streptomycin (100 μg/ml) at 37 °C with 5 % CO_2_. In each experiment, cells were cultured in 24-well plates, and then transient transfection was carried out using FuGene 6 reagent (Promega, Madison, WI), 2 μl of FuGene 6 was used per 1 μg of plasmids (reporter plasmids: 0.5 μg; expression plasmids: 0.5 μg) for each well. When different doses of an expression plasmid were used in an experiment, the total amount of transfected plasmid DNA was kept constant by addition of the empty control vector (pRc/RSV). Cells were harvested 48 h after the transfection and a luciferase assay was performed as described previously [17].

### Computer modeling of GATA3 structure

Three-dimensional structural modeling of wild-type and mutant GATA3 proteins was analyzed using SFAS provided by PDBj (http://pdbj.org/) [12]. The structural information of the wild-type GATA3 was derived from chain A of PDB entry 4hc7. Molecular graphics of the comparison of protein structures (residues 263–347) were created by UCSF Chimera which is a program to display molecular graphics of protein and nucleic acids developed by RBVI funded by the National Institutes of Health (http://www.cgl.ucsf.edu/chimera/) [13].

### Statistics

Samples in each group of transcription activation experiments were analyzed in triplicate. All of the experiments were performed more than twice to confirm the reproducibility, and the representative data are shown in the results. Data are expressed as means ± S.E. The differences between experimental values were analyzed by Student’s *t* test. Statistical analysis was performed using one-way ANOVA. *P*-values below 0.05 were considered significant.

## Discussion

In the present case, we identified a novel heterozygous missense mutation of *GATA3* in a Japanese family affected with HDR syndrome. This R299Q mutation induced an orientation change of the 2 zinc fingers and abolished its physiological function as a transcription factor.

The clinical manifestation of HDR syndrome is heterogeneous [3]. A triad of HDR symptoms is observed in 62.3 % of patients; 28.6 % of patients show only hypoparathyroidism and deafness, and 2.6 % of patients show only deafness and renal disease [18]. Hypoparathyroidism and hearing loss is the most common combination. Many different renal anomalies, with variable penetrance, are observed, including renal dysplasia, hypoplasia, aplasia, and vesico-ureteral reflux [3, 10, 11]. In addition, similar to our case, patients with HDR syndrome present with heterogeneous clinical manifestation even among individuals having the same mutation [10, 11]. The mechanism of heterogeneous symptoms has not been fully elucidated. In animal study, Grote et al. showed that the nephric duct-specific inactivation of *Gata3* leads to a wide spectrum of urogenital malformations through the glial cell line-derived neurotrophic factor/Ret signaling pathway [19]. Gata3 prevents ectopic ureter budding and premature differentiation of nephric duct cells. On the other hand, Gata3, which is also expressed in spiral ganglion neurons throughout their development, is essential for formation of the intricately patterned connections in the cochlea [20].

GATA3 belongs to a family of dual zinc finger transcription factors. Zinc fingers of the mammalian GATA proteins (GATA 1–6) have a Cys-*X*2-Cys-×17-Cys-*X*2-Cys structure (with X representing any amino acid residue), in which a single zinc ion is coordinated by 4 cysteine residues [7]. Its tertiary structure reveals 2 anti-parallel β-sheets, an α-helix, and a long loop. The α-helix binds into the major groove of DNA. In GATA3, ZnF2 is essential to the binding of the transcription factor to the consensus sequence (A/T)GATA(A/G) on the target gene promoter region, whereas ZnF1 is thought to stabilize this binding through its interaction with another cofactor, Friends of GATA (FOG) 2 [21, 22].

In this study, the 3-dimensional conformation of wild-type and mutant GATA3 was analyzed using SFAS, which runs several external programs for sequence alignment and structural modeling and organizes their results. Protein structure comparison was presented using another web application, UCSF Chimera, to construct high-resolution images. The structure of residues 263–347, ranging from ZnF1 to ZnF2, is displayed in Fig. 3. The configuration of the linker between the zinc fingers was affected severely by the mutation R299Q, which is located between the 2 zinc fingers. In addition, the mutation created a greater conformational change in ZnF1 than in ZnF2, probably because Arg-299 is located near the zinc ion of ZnF1. This Arg residue may stabilize a coordinate bond between the zinc ion and Cys residues, which are the crucial components of ZnF1. Substituting the basic amino acid (Arg) with neutral amino acid (Gln) may affect this stabilization and cause a change in the orientation in the protein structure. Furthermore, this conformational change spread into ZnF2. These results suggest that R299Q mutation impairs the function of both ZnF1 and ZnF2.

To date, the conformation of GATA3 mutant proteins has been investigated only in 3 cases of HDR syndrome [10, 11, 23]. Gaynor et al. showed that Thr272Ile, located in ZnF1, resulted in the loss of a polar side chain interaction between Thr-272 and Leu-274 [23]. This mutation changed the ZnF1 structure, leading to loss of DNA binding and FOG2 interactions. Ali et al. also showed that Leu348Arg could alter the transcriptional activity of GATA3 because of the substitution of a non-polar hydrophobic Leu residue for a positively charged Arg residue; this Leu 348 residue is located in ZnF2 and lies at the end of an α-helix which makes contact with DNA at the DNA major groove within the GATA motif of the promoter region [10]. Moreover, Chiu et al*.* analyzed the conformation change caused by Arg353Ser, which is located at the C-terminal DNA-binding region following ZnF2 (residues 318–342), using NNPredict software. This mutation was predicted to disrupt the helix turn composed of residues 355–358, because of the substitution of a basic for a polar, uncharged residue, which changed the angle between ZnF2 and the adjacent C-terminal tail [11]. However, our case is the first report of a conformation change caused by a mutation at the junction between the 2 zinc fingers; the amino acid substitution affected the conformation of both Zn fingers. Structure prediction model obtained by PDBj provides useful information about GATA3 mutant. Amino acids are classified into 4 groups such as nonpolar, polar, acidic, and basic amino acids. Substitution between amino acids belonging to different groups is likely to cause critical conformation change.

We also confirmed that R299Q was a loss-of-function mutation using an *in vitro* reporter assay system. GATA3/R299Q overexpression failed to increase the transcription activity of the GATA responsive element. In addition, our data showed that GATA3/WT activated *PTH* transcriptional activity, whereas GATA3/R299Q did not (Fig. 4b). This result is of interest, as there has been no previous direct evidence that GATA3 is a positive regulator of *PTH* expression. The consensus GATA3 binding element is not present in the 5′-promoter region of human *PTH*, suggesting that the observed enhancing effect of GATA3 is caused by an indirect pathway, rather than by the direct binding of GATA3. Further investigations are required to elucidate this molecular mechanism. Our results suggest that GATA3 plays an important role not only in the development of the parathyroid gland in fetus, but also in *PTH* regulation after birth.

## Conclusion

In this study, we identified a novel heterozygous missense mutation of *GATA3* in a Japanese family with HDR syndrome. We confirmed that the R299Q mutant is a loss-of-function mutation causing an extensive conformational change of 2 zinc fingers.

## Consent

Written informed consent was obtained from both patients for publication of this case report and all accompanying images. A copy of the written consent is available for review by the Editor of this journal.

## Abbreviations

CT: Computed tomography
GATA3: GATA binding protein 3
HDR: Hypoparathyroidism, deafness, and renal dysplasia
PTH: Parathyroid hormone
